# Pure Laparoscopic Left Hepatectomy for Regrowth of Mucinous Cystic Neoplasm of the Liver after Laparoscopic Deroofing

**DOI:** 10.1155/2022/4829153

**Published:** 2022-06-29

**Authors:** Hideki Kumagai, Akira Umemura, Hiroyuki Nitta, Hirokatsu Katagiri, Shoji Kanno, Daiki Takeda, Satoshi Amano, Koji Kikuchi, Kiyoharu Takashimizu, Masao Nishiya, Noriyuki Uesugi, Tamotsu Sugai, Akira Sasaki

**Affiliations:** ^1^Department of Surgery, Iwate Medical University, 2-1-1 Idaidori, Yahaba, Iwate, 028-3695, Japan; ^2^Department of Molecular Diagnostic Pathology, Iwate Medical University, 2-1-1 Idaidori, Yahaba, Iwate, 028-3695, Japan

## Abstract

**Background:**

Hepatic cystic lesions are common entities, most of which are simple hepatic cysts (SHCs). Mucinous cystic neoplasm of the liver (MCN-L) is a rare tumor characterized by ovarian-like stroma and accounts for <5% of all hepatic cysts. Distinguishing between SHCs and MCN-L is challenging because of the similarity in their imaging findings. Herein, we report a rare regrowth case of MCN-L after laparoscopic deroofing, treated with pure laparoscopic left hepatectomy. *Case Presentation*. A 63-year-old woman with a large hepatic cystic lesion and abdominal pain was referred to our hospital for surgical treatment. Computed tomography (CT) showed cystic lesions with septations arising from macrolobulations in the left medial segment. She underwent laparoscopic deroofing based on the diagnosis of SHCs; however, the final histopathological diagnosis was MCN-L. She chose observational follow-up, and MCN-L regrowth was detected on follow-up CT 6 months after the laparoscopic deroofing. We performed pure laparoscopic left hepatectomy for complete resection of the MCN-L. She had an uneventful postoperative course and no recurrence at the 5-year follow-up after the radical resection of the MCN-L.

**Conclusion:**

MCN-L is a rare entity, and accurate diagnosis with imaging modalities is greatly challenging. Laparoscopic hepatectomy for MCN-L should be considered as a strong alternative to secure safety and curability.

## 1. Introduction

Hepatic cystic lesions are common entities that occur in approximately 5–10% of the general population [1]; however, most of them are simple hepatic cysts (SHCs). In clinical practice, laparoscopic deroofing is usually performed for large and symptomatic SHCs [2].

On the other hand, mucinous cystic neoplasm of the liver (MCN-L) is a rare cystic liver tumor associated with ovarian-like stroma, according to the 2010 revision of the World Health Organization (WHO) classification [3]. In contrast to deroofing for SHCs, the treatment for MCN-L is oncological resection. Thus, distinguishing between MCN-L and SHCs preoperatively is important in selecting the optimal surgical method. However, it is greatly challenging because of the close similarity of their imaging findings [3, 4].

Herein, we report a rare regrowth case of MCN-L that required pure laparoscopic left hepatectomy in a patient who had previously undergone laparoscopic deroofing under the diagnosis of SHCs.

## 2. Case Presentation

**Figure 1:**
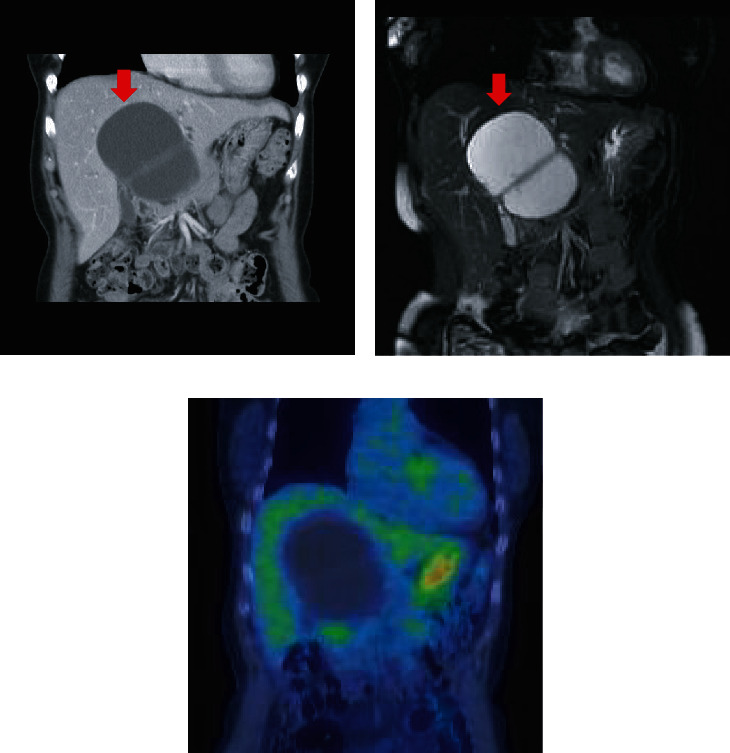
(a) Enhanced computed tomography and (b) T2-weighted magnetic resonance imaging show cystic lesions with septations arising from macrolobulations in the medial segment of the liver (red arrows). Uptake of fluorodeoxyglucose in the cystic lesions was not observed on positron emission tomography (c).

**Figure 2:**
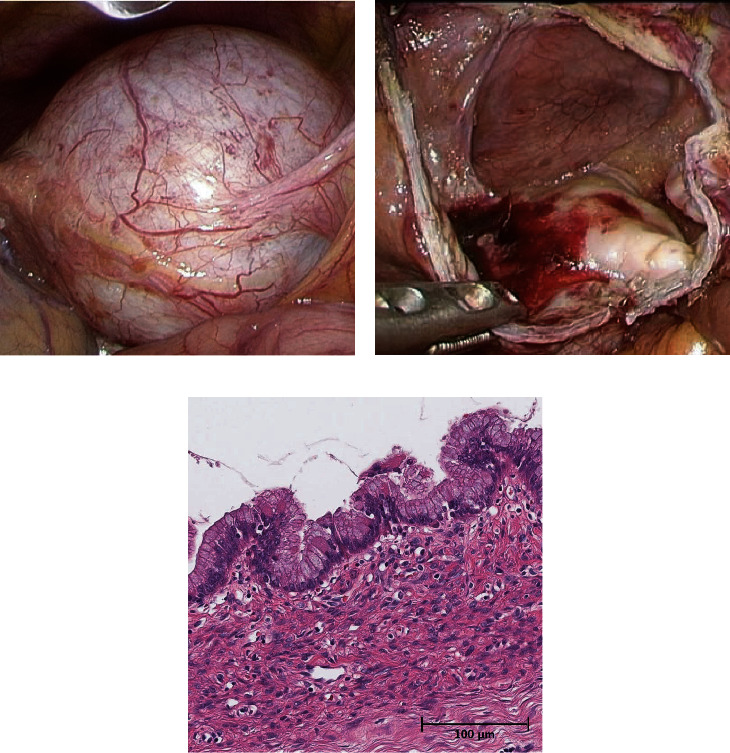
Intraoperative view before deroofing (a). A unilocular white cystic tumor was observed in the medial segment of the liver. Intraoperative view after deroofing (b). The lumen of cystic lesions was like velvet. Histopathological findings (hematoxylin and eosin staining, ×200 magnification) (c). The cyst wall was lined by mucin-producing atypical epithelium, which was surrounded by ovarian-like stroma. These findings indicated MCN-L.

**Figure 3:**
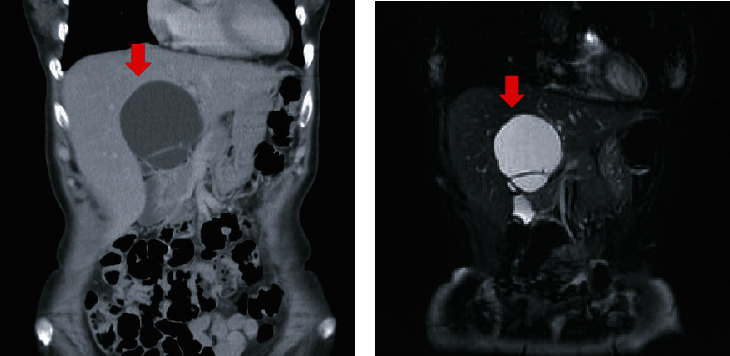
(a) Enhanced computed tomography and (b) T2-weighted magnetic resonance imaging at regrowth 6 months after deroofing showed cystic lesions with septations arising from the cyst wall without indentation (red arrows).

**Figure 4:**
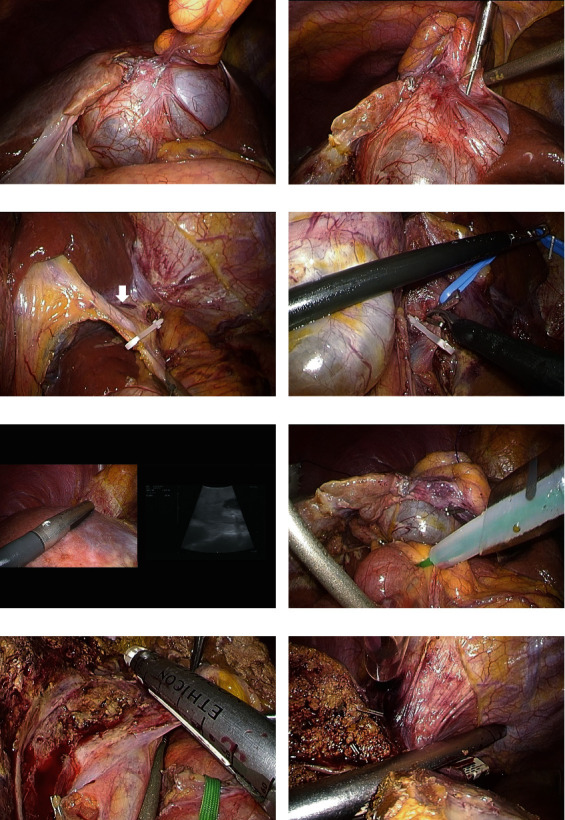
Surgical method is shown. A regrowth tumor was observed in the medial segment of the liver (a). Aspiration of cystic fluid was performed to secure a field of view (b). The left hepatic artery (white arrow) was diverged from the left gastric artery and clipped (c). The left portal vein was identified and divided after clipping (d). The transection line was set using intraoperative ultrasonography (e). Pringle's maneuver was performed prior to hepatic parenchyma transection (f). The (g) left hepatic duct and the (h) left hepatic vein were divided by a linear stapler.

A 63-year-old woman was followed up for a diagnosis of SHCs at a previous hospital. However, she complained of frequent abdominal pain. Enlargement of the hepatic cystic lesions was observed; thus, she was referred to our hospital for surgical treatment. Laboratory findings showed slightly elevated levels of serum liver enzymes as follows: aspartate aminotransferase, 40 IU/L; alanine aminotransferase, 42 IU/L; and gamma-glutamyl transpeptidase, 78 IU/L. The levels of serum tumor markers were also revealed, including those of carcinoembryonic antigen (CEA) and carbohydrate antigen 19-9 (CA19-9) which were also elevated; the levels of CEA and CA19-9 were 4.5 ng/mL and 125.8 U/mL, respectively. Enhanced computed tomography (CT) and magnetic resonance imaging (MRI) revealed cystic lesions with septations arising from macrolobulations in the left medial segment of the liver (Figures 1(a) and 1(b)). No dilatations of the intrahepatic bile duct and mural nodules in the cysts were not observed. Positron emission tomography revealed no fluorodeoxyglucose accumulation in the cystic lesions of the liver (Figure 1(c)). On the basis of these findings, the patient was diagnosed with SHCs and underwent laparoscopic deroofing of the cystic lesions (Figure 2(a)). Intraoperative findings showed that the cystic fluid was serous, not mucinous, suggesting SHCs. By contrast, the lumen of the cystic lesions was velvety, atypical of the characteristics of SHCs (Figure 2(b)). Histopathological examination revealed that the cystic wall was lined by an atypical mucin-producing epithelium surrounded by ovarian-like stroma; hence, the cystic lesions were diagnosed as MCN-L (Figure 2(c)). Watchful waiting was decided in accordance with the patient's choice. However, enhanced CT and MRI 6 months after the laparoscopic deroofing revealed regrowth of the cystic lesions, with septations arising from the cyst wall without indentation (Figures 3(a)–3(b)). As these findings were compatible with MCN-L regrowth, we performed pure laparoscopic left hepatectomy for complete resection of the MCN-L. The patient was placed in the supine position, and five trocars were inserted. The cystic tumor was in segment IV (Figure 4(a)), and no peritoneal dissemination was observed. Initially, we aspirated the cystic fluid to secure a good surgical field with careful attention to avoid fluid dissemination (Figure 4(b)). We identified the left hepatic artery diverging from the left gastric artery and left portal vein in the porta hepatis and clipped and divided them individually (Figures 4(c) and 4(d)). We then confirmed the demarcation line and the locations of the MCN-L, identified the middle hepatic vein using intraoperative ultrasonography, and set a transection line (Figure 4(e)). We transected the hepatic parenchyma using the intermittent Pringle maneuver (Figure 4(f)). The left hepatic duct and vein were divided using a linear stapler (Figures 4(g) and 4(h)). The resected specimen was placed in a plastic bag and extracted via an umbilical incision. The frequency and total duration of the Pringle maneuver were two times and 28 min 18 s, respectively. The operating time was 165 min, and blood loss was 7 mL. Histopathological examination revealed that the tumor was an MCN-L with low-grade dysplasia and that the resected MCN-L specimen had a sufficient surgical margin. The patient had an uneventful postoperative course and was discharged on postoperative day 9. She had no recurrence at the 5-year follow-up after the radical resection of the MCN-L.

## 3. Discussion

Hepatic cystic lesions are frequently encountered in clinical practice and estimated to occur in approximately 5–10% of the general population [1], mostly as SHCs. However, MCN-L is rare, accounting for <5% of all hepatic cystic lesions [1]. MCN-L was formerly referred to as biliary cystadenoma but was newly defined as a tumor associated with ovarian-like stroma according to the 2010 revision of the WHO classification [3]. As a side note, malignant MCN-L is extremely rare, accounting for a mere 2% of all MCN-Ls [5].

MCN-L is difficult to diagnose preoperatively. In particular, preoperative distinction between MCN-L and SHCs is greatly challenging [3, 4]. The discrepancy rate between preoperative diagnosis with imaging modalities and final diagnosis with histopathological examination using specimens resected using surgical procedures reached ≥30% [6]. Boyum et al. reported several imaging features for distinguishing between MCN-L and SHCs: septations arising from the cyst wall without indentation suggest MCN-L, whereas unilocular cystic lesions or septations arising only from macrolobulations suggest SHCs [4]. In the present case, the initial enhanced CT and MRI revealed cystic lesions with septations arising from macrolobulations in the medial segment of the liver. These findings suggested SHCs, and the patient underwent laparoscopic deroofing. However, the histopathological diagnosis was MCN-L, in contrast to the preoperative diagnosis of SHCs. Another hepatic cyst-forming tumor mimicking MCN-L is intraductal papillary neoplasm of the bile duct (IPNB) [3]. IPNB is a counterpart of intraductal papillary mucinous neoplasm of the pancreas and is characterized by papillary growth in the intrahepatic or extrahepatic bile duct. Both MCN-L and IPNB form cystic lesions in the liver because of mucin production [3]. Communication with and dilatation of the bile ducts are comparative characteristics of IPNB [3], but these entities are also present, albeit rarely, in MCN-L [7]. Therefore, discrimination between these tumors on imaging modalities is often difficult, and many cases had been diagnosed definitively on the basis of pathohistological findings with or without the presence of ovarian-like stroma.

The optimal operative method for hepatic cystic lesions is difficult to determine because of the difficulty of preoperative diagnosis, as mentioned earlier. Some surgical considerations for hepatic cystic lesions are as follows: cyst size > 100 mm, enlargement during surveillance, suspicion of malignancy, and associated symptoms including abdominal pain, oppressive feeling, and fever [5]. In clinical practice, laparoscopic deroofing is typically used for large and symptomatic SHCs [2]. The treatment of MCN-L involves oncological resection. As most MCN-L cases are benign, the prognosis after complete resection is excellent [8]. However, deroofing or fenestration, which is generally performed for SHCs, is not sufficient for MCN-L and leads to recurrence at 90–100% probability [9]. In the present case, the patient experienced early recurrence of MCN-L at 6 months after laparoscopic deroofing. The use of laparoscopic surgery for hepatic tumors, whether benign or malignant, has been gradually increasing, and the surgical procedure has been stylized. We also perform laparoscopic hepatectomy for complex hepatic tumors [10], as it confers a lower risk of major morbidity and leads to a shorter hospital stay than open hepatectomy [11]. Laparoscopic surgery is a valid procedure for MCN-Ls in terms of minimizing surgical invasion because most MCN-L cases are benign [5]. To our knowledge, the literature on laparoscopic left hepatectomy as treatment for MCN-L is limited [12, 13]. Enucleation is a less-invasive procedure, but it is not appropriate in cases suspected of malignancy. In the present case, the patient had early tumor regrowth, which suggested the possibility of malignancy. Thus, we decided to perform pure laparoscopic left hepatectomy for oncological resection to prevent recurrence. As a result, the patient had no complications or recurrence. Laparoscopic hepatectomy can be performed safely in well-experienced institutions and can be an effective treatment for MCN-L.

## 4. Conclusion

MCN-L is a rare entity, and accurate diagnosis using imaging modalities is challenging. When examining patients with hepatic cystic lesions, the possibility of MCN-L should be considered, and MCN-L should be distinguished from other hepatic cystic lesions as much as possible to determine the optimal operative procedure. Laparoscopic surgery for MCN-L is safe and reasonable to perform in experienced facilities.
